# Drug targeting of aminopeptidases: importance of deploying a right metal cofactor

**DOI:** 10.1007/s12551-024-01192-8

**Published:** 2024-04-24

**Authors:** Saleem Yousuf Bhat

**Affiliations:** University of Pennsylvania, Philadelphia, 19104 USA

**Keywords:** Aminopeptidases, Hydrolases, MetAPs

## Abstract

Aminopeptidases are metal co-factor-dependent hydrolases releasing N-terminal amino acid residues from peptides. Many of these enzymes, particularly the M24 methionine aminopeptidases (MetAPs), are considered valid drug targets in the fight against many parasitic and non-parasitic diseases. Targeting MetAPs has shown promising results against the malarial parasite, *Plasmodium*, which is regarded as potential anti-cancer targets. While targeting these essential enzymes represents a potentially promising approach, many challenges are often ignored by scientists when designing drugs or inhibitory scaffolds against the MetAPs. One such aspect is the metal co-factor, with inadequate attention paid to its role in catalysis, folding and remodeling of the catalytic site, and its role in inhibitor binding or potency. Knowing that a metal co-factor is essential for aminopeptidase enzyme activity and active site remodeling, it is intriguing that most computational biologists often ignore the metal ion while screening millions of potential inhibitors to find hits. Ironically, a similar trend is followed by biologists who avoid metal promiscuity of these enzymes while screening inhibitor libraries in vitro which may lead to false positives. This review highlights the importance of considering a physiologically relevant metal co-factor during the drug discovery processes targeting metal-dependent aminopeptidases.

**Graphical abstract:**

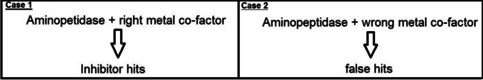

## Introduction

Aminopeptidases are metal-dependent enzymes that catalyze the cleavage of amino acids from the amino terminal end (N-end) of proteins or peptides, that is, they are exopeptidases (Bradshaw 2013). They are widespread across living systems and are found in different subcellular organelles and cytosol (Kim et al. 2009) and can also be attached to the membrane (Cristofoletti et al. 2006). Aminopeptidases are utilized in fundamental cellular processes such as protein maturation and turnover (Lowther and Matthews, 2000; Sanderink et al. 1988). However, not all of these peptidases are zinc-dependent metalloenzymes (Makarova and Grishin 1999).

Some of these enzymes are monomeric (Bhat et al. 2018), and others exist and function as oligomers of multiple subunits, usually dimers (Bhat and Qureshi 2020) or higher order oligomers (Timm et al. 2017; Michalska et al. 2005). Sequences and structures of numerous aminopeptidases such as alanine aminopeptidases, methionine aminopeptidases, and human endoplasmic reticulum aminopeptidase 1 (ERAP1) are housed in the protein data bank (PDB). Gene sequences of aminopeptidases across living beings deduced directly or from cDNAs demonstrate crucial amino acid arrangement homologies in chemically or catalytically significant positions such as residues involved in metal co-factor binding (Bhat et al. 2018; Bhat et al. 2020a, b). Some significant aminopeptidases are the M18 aspartyl aminopeptidase of humans involved in blood pressure regulation (Chaikuad et al. 2012) and the neutral aminopeptidases of parasite *Plasmodium falciparum*, involved in host hemoglobin catabolism (Dalal and Klemba 2007).

Aminopeptidases are widely distributed across living systems and carry out crucial functions such as protein maturation and peptide degradation (Gonzales and Robert-Baudouy 1996). This role in protein maturation (Frottin et al. 2006) includes hormonal and non-hormonal peptide degradation (Mizutani et al. 2013). Consequently, many ailments are related with hindered proteolytic function (López-Otín and Bond 2008).

Aminopeptidases are grouped:

1. On the basis of number of amino acids cleaved from the N-terminal end. Enzymes that successively excise the amino terminal amino acids from protein and peptide substrates are termed as aminopeptidases, whereas aminodipeptidases hydrolyze dipeptides from the NH2-end. Similarly, aminotripeptidases hydrolyze tripeptides from amino terminal of peptide substrates (Taylor 1993; Evrin et al. 2009).
2. Depending upon the relative effectiveness with which different amino acid residues are liberated, for instance, alanine aminopeptidases most effectively release Ala from peptides even though it can hydrolyze other residues less efficiently. Similarly, aspartyl aminopeptidase releases acidic residues aspartate and glutamate more efficiently while methionine aminopeptidase releases methionine (Lowther and Matthews 2000; Taylor 1993).
3. On the basis of their location in cells. A few aminopeptidases are secretory proteins (Squire et al. 1991), and others are cytosolic (Bhat and Qureshi 2020) or membrane bound (Simmons and Orawski 1992). Some peptidases are found in organelles, for example, lysosomes (Ivry et al. 2019) or mitochondria (Taylor 1993).
4. According to metal dependence of these proteins. As metals such as Zn(II), Mn(II), Fe(II), or Co(II) are often the metal activators of these enzymes, any enzyme activated highly by any divalent cation assumes the name starring the metal activator, for instance, Zn(II)-activated aminopeptidase (Taylor 1993; Calcagno and Klein 2016).
5. Depending upon the pH at which optimal activity is obtained. As such, aminopeptidases can be acidic, basic, or neutral peptidases. The usual pH of optimal activity however tends to be neutral or physiological (Taylor 1993).

The major information center of aminopeptidases is the MEROPS database (Rawlings and Barrett 1999) which bears information about almost all studied peptidases, be it metal-dependent aminopeptidases or other proteases along with the known inhibitors. This database also offers both family and clan information, on classification and nomenclature of the peptidases. Unlike conventional classification systems on peptidases, the MEROPS database uses both a hierarchical and structure guided classification. As such, every peptidase is assigned to a family based on similarities in amino acid sequence, while peptidase families possessing high homology with one another are assigned to the same clan (https://www.ebi.ac.uk/merops/).

**Fig. 1:**
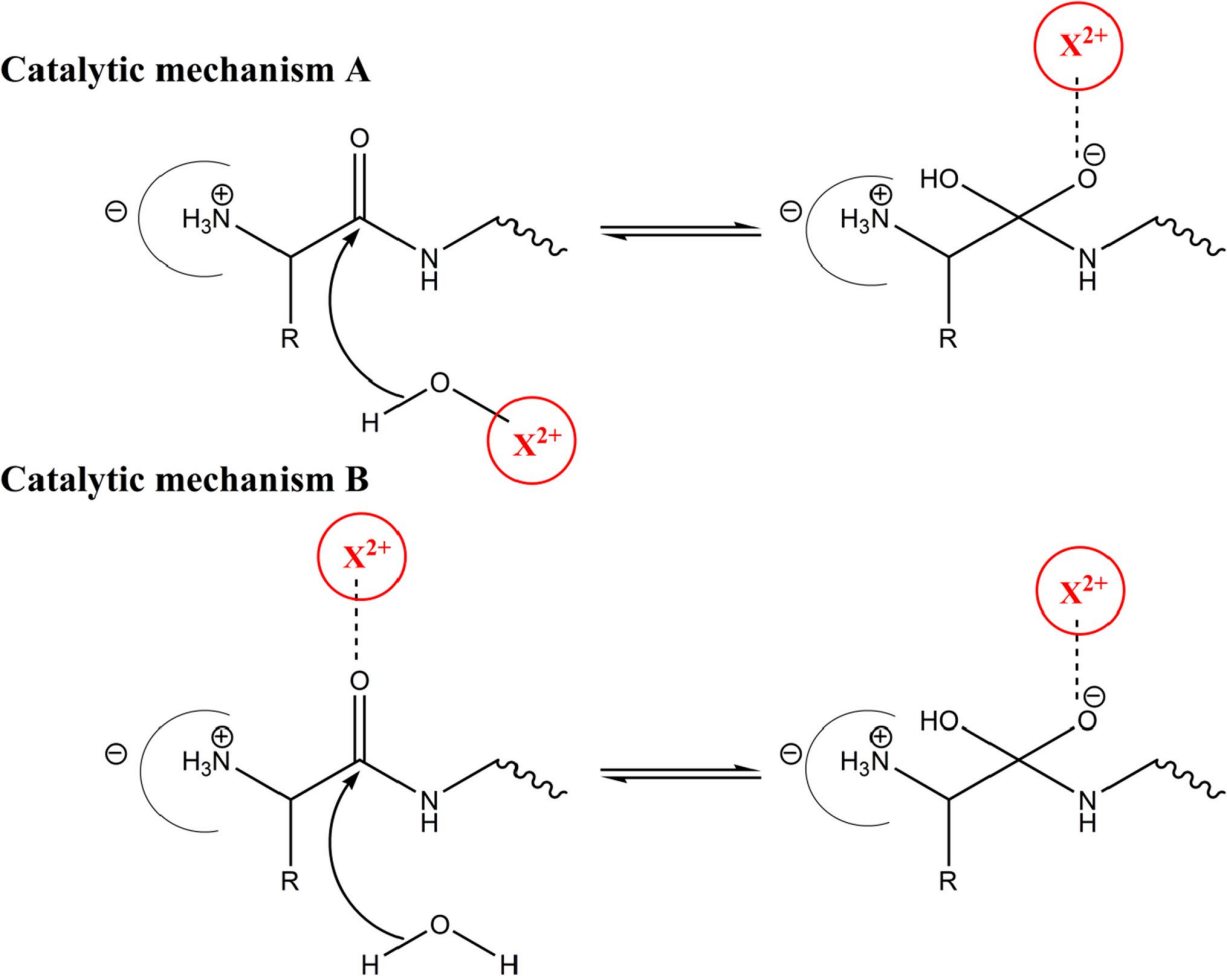
Role of metal-cofactor in the catalytic mechanism of aminopeptidases: stabilization of reactive hydroxide ion (catalytic mechanism **A**), facilitation of the nucleophilic attack of a water molecule (catalytic mechanism **B**). *X*^2+^ (red) represents a divalent metal co-factor (Zn^2+^, Mn^2+^, Fe^2+^) (Chaikuad et al. 2012; Mucha et al. 2010; Bennett and Holz 1997)

In order for catalysis to take place, aminopeptidases require metal co-factors which interact with the catalytic residues (Bhat and Qureshi 2020), (Addlagatta et al. 2005). Another feature is that the metal co-factor is neighbored by a hydrophilic shell that is immersed in a broader hydrophobic environment (Timm et al. 2017). The active site composition and structures suggest the feasibility of two major catalytic reaction pathways involving metal co-factors (Mucha et al. 2010). Firstly, a co-factor may stabilize a highly reactive hydroxide ion, which in turn ensures the availability of an activated nucleophile for catalysis at physiological pH (Mucha et al. 2010; Bhat and Qureshi 2021). Secondly, the positively charged metal co-factor may act as an electrophilic catalyst, complexing an oxygen atom of the scissile peptide bond and enabling the water molecule’s nucleophilic attack (Chaikuad et al. 2012; Mucha et al. 2010; Bhat and Qureshi 2021; Bennett and Holz 1997) (Fig. 1).

## Methionine aminopeptidases and the importance of metal co-factor in drug discovery

Methionine aminopeptidases (MetAPs) are a highly conserved and ubiquitous class of metal co-factor-dependent aminopeptidases that release the N-terminal initiator methionine from nascent polypeptides either during translation or when a complete polypeptide is synthesized (Bhat et al. 2018). These hydrolytic enzymes are placed with the M24 peptidases according to the classification available in the MEROPS peptidase database (Rawlings and Barrett 1999). It is important that the initiator methionine residue is excised to allow the occurrence of further post-translational modifications of new polypeptides viz. myristoylation, localization, proper protein folding, or activity in eukaryotes (Bhat et al. 2018; Addlagatta et al. 2005; Gonzales and Robert-Baudouy 1996). MetAPs have also been implicated in the determination protein half-life as a function of the N-end rule (Gonzales and Robert-Baudouy 1996). To excise the methionyl residue, MetAPs usually require a small uncharged residue such as glycine, alanine, or valine at the penultimate position of the incipient polypeptide chain (Giglione et al. 2004). MetAPs require divalent metal cations as cofactors for catalysis, and many studies on MetAPs demonstrate Co(II), Fe(II), and Mn(II) to be the most favored divalent metal activators (Bhat et al. 2020a, b).

**Fig. 2:**
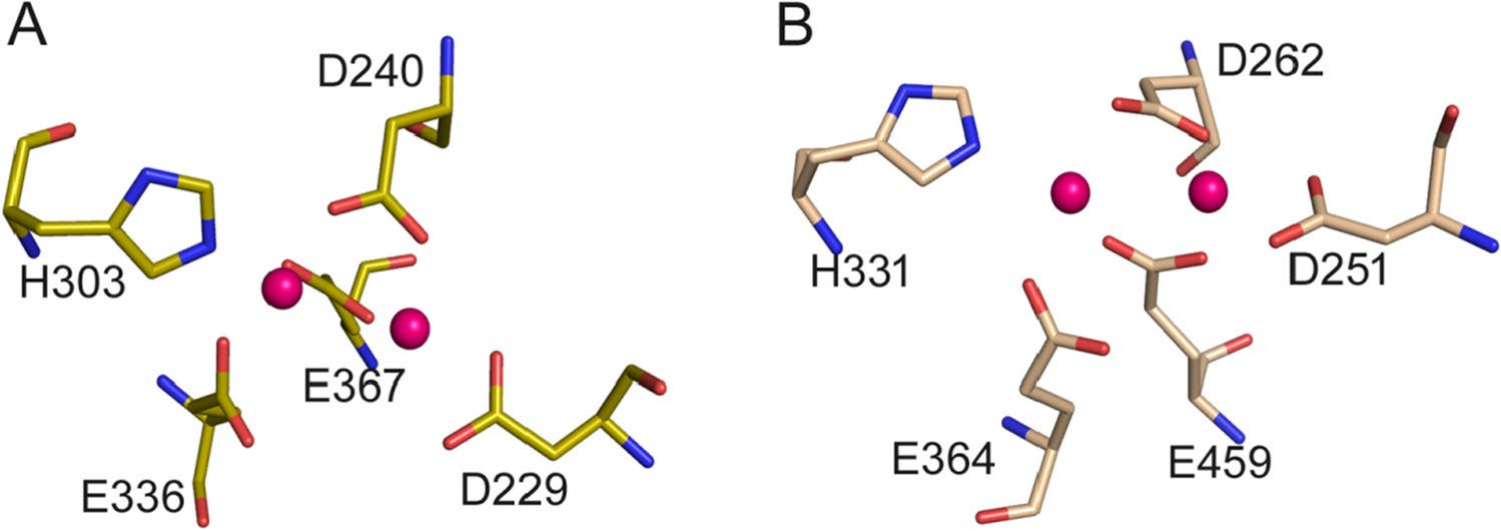
Conserved catalytic sites of human MetAP1 (**A**) and MetAP2 (**B**) from the PDB codes 4HXX and 1BN5, respectively. Pink circles represent the two metal co-factors

MetAPs are grouped into two classes: type I (MetAP1) and type II (MetAP2), with the distinctive insertion of almost 60 amino acids in MetAP2 at the C-end of the catalytic domain (Addlagatta et al. 2005). MetAPs found in prokaryotic bacteria and archaea possess the minimal catalytic domain while MetAPs of eukaryotes carry extra N-terminal domains, for example, the presence of N-terminal zinc-finger domain found in human MetAPs (Liu et al. 1998). These enzymes have a conserved active site (Fig. 2).

Eukaryotic genomes encode for both isoforms of MetAPs while prokaryotic express only one isoform: type I in bacteria and type II in archaea (Calcagno and Klein 2016). A past report on the expression of two isoforms in eukaryotes indicates MetAP1 to be constitutively expressed and is thus considered as a house-keeping protein (Hu et al. 2006). MetAP2 expression is directed by extracellular signals and cell cycle status (Chatterjee et al. 1998). The deletion of prokaryotic MetAP1 is reported to be lethal (Chang et al. 1989). Eukaryotes carry both types of MetAPs and tend to develop lethal phenotypes if either one or both MetAP genes are deleted or the protein products inactivated (Li and Chang 2006). Therefore, a lot of attention was given to MetAP biochemistry and development of specific small-molecule inhibitors definitively targeting these enzymes to contain parasitic diseases (Rosenthal 1998), obesity (Joharapurkar et al. 2014), and cancer (Frottin et al. 2016).

Due to their essential nature, these enzymes have been dubbed as promising druggable candidates against many parasitic diseases, including the neglected tropical diseases (NTDs) (Bhat et al. 2018). Many groups have therefore designed chemical libraries to find new inhibitors and employed computational resources to screen millions of potential inhibitors (Bhat et al. 2020a, b) (Chen et al. 2006). Such chemical libraries or potential inhibitors are often chemical derivatives of substrate analogs made to ensure that any new inhibitor binds the catalytic site, by potentially competing with the substrate to bind to the catalytic core (Heinrich et al. 2019; Hirst et al. 2020). Many of these studies have directly employed potent substrate mimicking analogs as prodrugs, producing potent inhibition (Bhat et al. 2018; Wang et al. 2003a, b). Others have used the structural information to find new inhibitors (Hirst et al. 2020). One issue with the use of substrate mimicking species is their lack of specificity (Qvit et al.^2017^). In other words, a substrate mimicking inhibitor binds to and inhibits many aminopeptidase families (Bhat and Qureshi 2020, 2021). For instance, the peptide mimicking substrates (peptidomimetics) such as bestatin, actinonin, and amastatin are potent inhibitors of M20, M17, and M18 aminopeptidases (Bhat and Qureshi 2020, 2021; Michalska et al. 2005). Because there is no one true substrate mimic of any aminopeptidase, new inhibitor discovery based on the principle of substrate mimicking has remained elusive (Giastas et al. 2019). However, new drug discovery efforts may be driven by the different amino acid composition near the catalytic pocket which often changes the size and physicochemical properties of the catalytic site and enables new inhibitor discovery (Bhat et al. 2020a, b). Notably, aminopeptidases may not always be targeted with only catalytic or competitive inhibitors. Both biochemical and structural data suggest aminopeptidases can be inhibited by drugs targeting regions other than the catalytic site (Bhat et al. 2020a, b; Schiffmann et al. 2005).

Being metal co-factor-dependent enzymes, aminopeptidases are inactive in the absence of a metal co-factor (Bhat and Qureshi 2020; Gonzales and Robert-Baudouy 1996; McGowan et al. 2009). The molecular, structural, and biochemical information available till date implicates two metal co-factors in catalysis (Bhat and Qureshi 2020; Modak et al. 2015; Hu et al. 2006). While many studies have indicated one metal being a physiological activator, the true co-factor responsible for activity for all known aminopeptidases has been a subject of some controversy and also an open debate (Malcolm et al. 2021; Wang et al. 2003a, b; Marschner and Klein 2015). A striking feature of the two metal co-factors is that one may be readily replaceable while the other is almost permanently bound to the catalytic site (Modak et al. 2015; Dutoit et al. 2019). This is particularly true for leucine aminopeptidases (Modak et al. 2015).

**Fig. 3:**
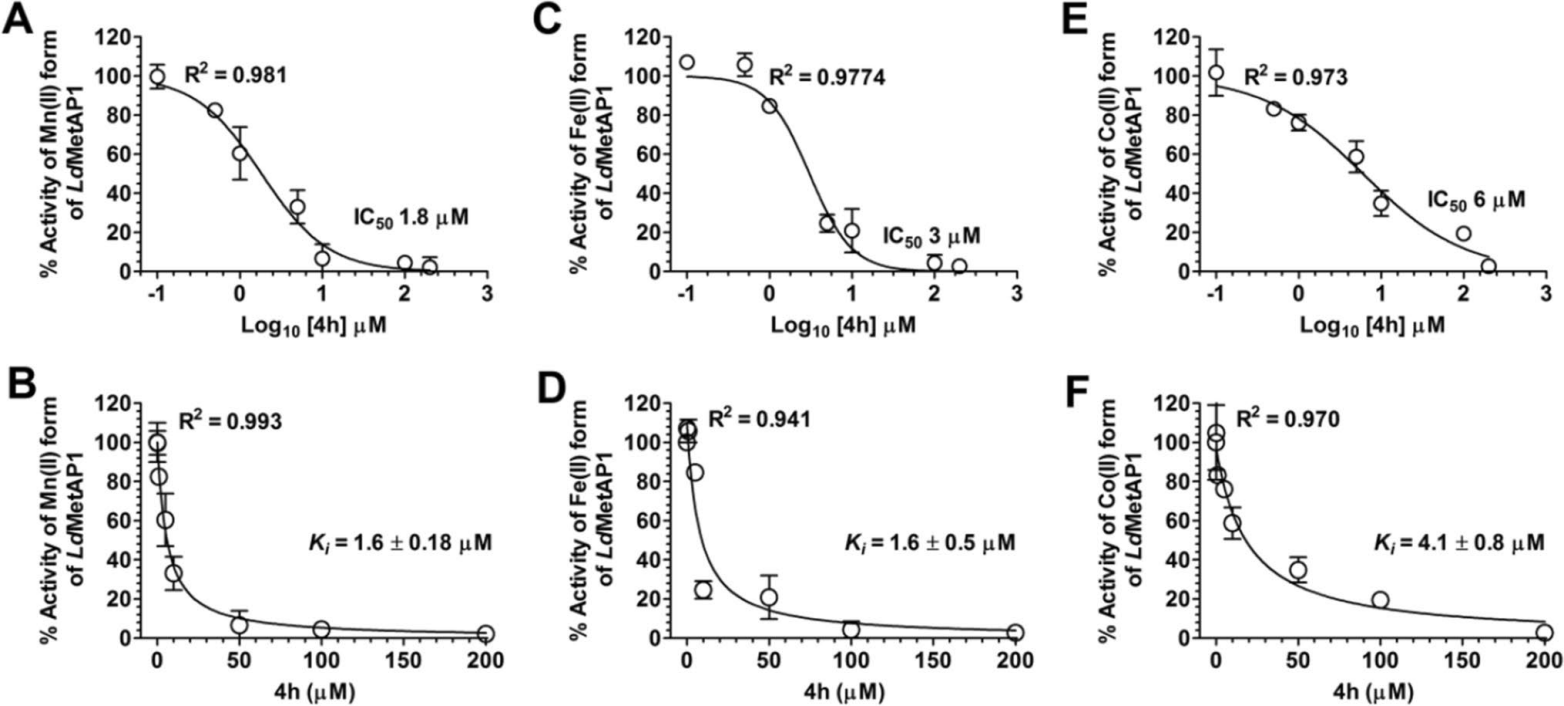
Metal-dependent aminopeptidase inhibition of the small-molecule inhibitor 4 h highlighting that different co-factors impact inhibitor potency. The figure is adapted with permission from Bhat et al. (2021)

Another aspect of such metal co-factor requirement is the differential activity observed with different metal chlorides, possibly due to the differences in the metal characteristics of transition metals in relation to size and charge (Harty et al. 2019; Bowen and Dupureur 2003; Bhagi-Damodaran et al. 2017). Whether this means an aminopeptidase may have multiple co-factors for catalysis in vivo is unknown. For instance, cobalt chloride readily drives the catalytic activity of almost every known aminopeptidase (Bhat and Qureshi 2020; Bhat et al. 2020a, b); however, studies show that a metal co-factor other than cobalt may be a true metal co-factor physiologically (D’souza and Holz 1999; Bhat and Qureshi 2021). This is true for methionine aminopeptidases (MetAPs), particularly the type I MetAP, which is regarded as an iron-dependent enzyme although it exhibits tremendous catalytic activity with cobalt supplementation in vitro (Bhat et al. 2020a, b). It is my contention that many studies conducted for rational drug design, inhibitor discovery targeting MetAPs have either avoided the role of metal in the inhibitor potency or considered metal which may not be the physiologically relevant (Chowdhury et al. 2012; Weako et al. 2020; Arya et al. 2015). Both these approaches may be scientifically limited as the aim is always to inhibit an active enzyme, and these enzymes are active only when supplemented with a metal co-factor. As such, it becomes necessary to explicitly recognize the physiologically relevant metal-cofactor in inhibitor or drug discovery trials. Many studies based on screening chemical libraries have only used a single metal co-factor when it is possible that physiologically, an enzyme may have more than one co-factor for activity. Thus, testing the potency of new hits with multiple metal co-factors assumes importance, and it may be more appropriate to use or supplement the assay buffer with a physiologically relevant metal chloride when screening chemical libraries. With regard to MetAPs, it is widely accepted that type I MetAPs are druggable candidates and are Fe(II)-dependent enzymes as experimentally, it was shown that when type I MetAP was overexpressed, only the quantity of Fe(II) increased suggesting that Fe(II) is indeed the physiological co-factor of type I MetAPs (D’souza and Holz 1999). A recent study on type I MetAP from *Leishmania* underscored the role of metal in inhibitor potency as the authors observed differences in potency (Fig. 3) upon using different metal co-factors (Bhat et al. 2020a, b). This suggests that any drug trial focused on MetAPs should consider the inhibitor-enzyme complex formation in the presence of co-factor as metal co-factors play important role in potency for most classes of aminopeptidase inhibitors reported till date. This is now particularly important as aminopeptidases are gaining importance as drug targets due to their immense importance as host factors for many potentially deadly pathogens like corona viruses in the contemporary world (Wang et al. 2021).

As metal co-factor is important for catalysis, a feasible way to inactivate an aminopeptidase could be the sequestration of metal co-factor with a small molecule. Some inhibitors of aminopeptidases like EDTA typically sequester the catalytic metal co-factors and cause enzyme inhibition (Shapiro et al. 2011). Thus, sequestering a metal ion may be a potential strategy to inhibit aminopeptidases. Importantly, any new inhibitor showing metal-dependent inhibition should be characterized for its mechanism of action.

It is interesting that metals induce oligomerization in some aminopeptidases. Such reports have recently emerged for M24 and M17 aminopeptidases (Dutoit et al. 2019; Malcolm et al. 2021). Whether the metal-induced oligomerization is necessary for some aminopeptidase function in vivo is not clear. But if it is, then it becomes increasingly important to use the physiologically relevant metal in the biochemistry-guided aminopeptidase assays or the inhibitor screening.

In my opinion, it is wrong to call an aminopeptidase a cobalt-dependent aminopeptidase just because cobalt could be fitted into the metal density in the catalytic site. It is to be understood that if you see cobalt in the crystal structure, then it is the metal used while screening crystal conditions or the metal present in the mother liquor or soaking solution which leads to the presence of a certain metal in the crystal lattice. However, it may not be the real metal co-factor involved in the catalysis physiologically. In other words, the inability to get the structure solved in the presence of the physiological metal co-factor does not imply that it is not the co-factor used by enzyme for catalysis. This should be particularly true for zinc-dependent aminopeptidases as growing crystals with zinc is notoriously difficult due to its protein precipitation tendencies (general experience with aminopeptidases). Sometimes, the aminopeptidases may take up the metal co-factor from the expression system itself, and in such a scenario, there is a chance that enzyme has taken up the most suitable metal co-factor responsible for its activity. Consequently, a crystal structure of such a purified protein would mean that the protein was crystallized with its native metal, and thus, the structure may represent an active aminopeptidase structure (Modak et al. 2015; Xu et al. 2012). The importance of such a structure is immense as having the right metal at the active site would mean the right sort of folding of the catalytic site which can be used for inhibitor screening computationally or co-crystallized with the inhibitor hits found via chemical library screenings. Interestingly, the crystal structures of cobalt and manganese forms of methionine aminopeptidase (PDB codes: 2B3H and 4FLI) do not appear to differ suggesting different metal properties modulated protein inhibition during the formation of a metal-protein-inhibitor ensemble.

## Conclusions

Metal-dependent aminopeptidases particularly the methionine aminopeptidases are receiving significant attention as potential drug targets in the fight against many parasitic and non-parasitic diseases. As such, research focusing on the discovery of new inhibitor molecules targeting these enzymes is in full swing. However, in order to produce small molecules with inhibitory activity, it is essential that the active enzyme be supplemented with the physiologically relevant metal co-factor in high throughput screening or drug discovery trials. This gains importance as scientific evidence suggesting a key role of metal not just in catalysis but also in inhibitor potency of many classes of inhibitors is emerging.
